# Pre‐corticosteroid delusional psychosis and olanzapine‐responsive behavioral dyscontrol in autoimmune glial fibrillary acidic protein astrocytopathy: A case report

**DOI:** 10.1002/pcn5.70373

**Published:** 2026-07-07

**Authors:** Tomomi Sugisaki, Yoshiteru Sato, Hiroaki Fujita, Yasushi Kawamata, Norio Sugawara, Keisuke Suzuki, Norio Yasui‐Furukori

**Affiliations:** ^1^ Department of Psychiatry Dokkyo Medical University Mibu Tochigi Japan; ^2^ Department of Neurology Dokkyo Medical University Mibu Tochigi Japan

**Keywords:** autoimmune encephalitis, delusion, GFAP astrocytopathy, olanzapine, organic psychosis

## Abstract

**Background:**

Autoimmune glial fibrillary acidic protein (GFAP) astrocytopathy is an immunotherapy‐responsive inflammatory central nervous system disorder. Psychiatric manifestations include delirium, cognitive or behavioral change, apathy, depressive symptoms, and anxiety; however, frank delusional psychosis before corticosteroid exposure is rarely characterized. Timing is critical because corticosteroids can themselves cause psychosis.

**Case Presentation:**

A 42‐year‐old man with no previous psychotic disorder developed a progressive subacute syndrome over several months, beginning with headache and followed by impaired fine motor function, dysarthria, dysphagia, tremor, and cognitive decline. Cerebrospinal fluid (CSF) showed inflammation, and CSF GFAP‐immunoglobulin G (IgG) was confirmed by qualitative cell‐based and tissue‐based assays. Before corticosteroid pulse therapy or oral prednisolone, his family observed a new persecutory delusion that someone was outside the window. Immunotherapy was led by neurologists, and psychiatric symptoms were assessed by psychiatrists through consultation and later psychiatric hospitalization. After corticosteroid therapy, neurological symptoms partially improved, but persecutory ideas, grandiosity, insomnia, irritability, aggression, and escape behavior became prominent. Prednisolone was continued because of the autoimmune neurological disease. Olanzapine was titrated to 15 mg/day. Behavioral dyscontrol improved markedly despite continued prednisolone, and physical restraint was no longer required; however, residual grandiose delusional ideas persisted.

**Conclusion:**

This case suggests that delusional psychosis can be an early manifestation of autoimmune GFAP astrocytopathy. Because the first delusion preceded corticosteroid exposure, steroid‐induced psychosis was excluded as the mechanism of onset. Olanzapine may stabilize severe behavioral dyscontrol while necessary corticosteroid therapy is maintained, although residual delusions may persist.

## BACKGROUND

Autoimmune glial fibrillary acidic protein (GFAP) astrocytopathy is a rare autoimmune inflammatory disorder of the central nervous system associated with GFAP‐immunoglobulin G (IgG), particularly in cerebrospinal fluid (CSF), and typically presents as meningoencephalitis, encephalomyelitis, or meningoencephalomyelitis.^1^, ^2^, ^3^, ^4^, ^5^, ^6^, ^7^, ^8^ Reported manifestations include headache, fever, encephalopathy, tremor, ataxia, dysphagia, visual symptoms, cognitive impairment, and psychiatric or behavioral symptoms.^1^, ^2^, ^3^, ^4^, ^5^, ^6^, ^7^, ^8^, ^9^, ^10^

The psychiatric manifestations reported in previous GFAP astrocytopathy literature have generally been heterogeneous and often nonspecific. Shan et al. summarized psychiatric disorders and behavioral symptoms within encephalitic presentations.^3^ Dubey et al. noted psychiatric symptoms in a prospective GFAP astrocytopathy cohort, but detailed delusional content was not systematically characterized.^4^ Kunchok et al. grouped psychiatric symptoms with encephalitic features such as delirium, tremor, and seizures.^8^ Tomczak et al. described a patient with progressive somnolence, apathy, anxiety, and subsequent acute delusional psychosis with parkinsonism.^9^ A recent systematic review and meta‐analysis confirmed psychiatric symptoms among recognized clinical phenotypes, but individual psychotic phenomenology and antipsychotic response were not consistently detailed.^10^ Thus, cognitive decline, apathy, depressive symptoms, anxiety, delirium, and nonspecific behavioral change are better represented in the literature than carefully timed, frank delusional psychosis.^3^, ^4^, ^8^, ^9^, ^10^, ^11^, ^12^

Corticosteroids are a mainstay of treatment for GFAP astrocytopathy, but they can also cause insomnia, mood symptoms, delirium, and psychosis.^13^, ^14^, ^15^, ^16^ Therefore, psychosis appearing during treatment may be misattributed to corticosteroids. We report a case of autoimmune GFAP astrocytopathy in which persecutory delusions appeared before corticosteroid exposure and in which severe behavioral dyscontrol improved after olanzapine while prednisolone was continued.

## CASE PRESENTATION

A 42‐year‐old man had no history of psychotic disorder. Exact calendar dates are omitted to protect patient privacy; the relative sequence of events is summarized in Table 1. His illness evolved over several months rather than emerging simultaneously. Headache developed first. Approximately 2 months later, he developed difficulty with fine motor tasks and impaired work performance. During the following several months, dysarthria, dysphagia, tremor, reduced appetite, weight loss, psychomotor slowing, and cognitive decline became apparent.

**Table 1:** Relative clinical timeline and treatment response.

Approximate interval	Clinical phase	Neurological and immunological findings	Psychiatric findings	Treatment and outcome
Month 0	Initial symptom onset	Subacute headache	No psychotic symptoms recognized	No corticosteroid exposure
Approximately Month 2	Progression of neurological symptoms	Fine motor impairment and impaired work performance	No established psychotic symptoms	Neurological evaluation was pursued
Approximately Months 4–5, before corticosteroids	Autoimmune encephalitis suspected	Dysarthria, dysphagia, tremor, cognitive decline; CSF inflammation; CSF GFAP‐IgG positivity confirmed by CBA and TBA at 1:4 dilution	New persecutory delusion that someone was outside the window; possible hallucinatory experiences noted during early psychiatric assessment	No corticosteroid exposure before the first delusion; chronology rules out steroid‐induced onset
Shortly thereafter	After immunotherapy	Intravenous corticosteroid pulse therapy followed by oral prednisolone; maximum documented oral prednisolone dose 60 mg/day; partial improvement of tremor and dysarthria	Persecutory delusions, suspiciousness, grandiosity, insomnia, irritability, emotional lability	Prednisolone continued/tapered according to neurological status
Several months after onset	Psychiatric hospitalization	No clear infection, hepatic dysfunction, or severe metabolic disturbance explaining psychosis; autoimmune diagnosis remained central	Agitation, disorganized behavior, aggression, escape behavior, poor impulse control	Olanzapine titrated to 15 mg/day while prednisolone was continued
Within the psychiatric hospitalization	Outcome	Neurological follow‐up continued	Agitation, aggression, escape behavior, and need for restraint improved markedly; residual grandiose delusional ideas persisted	Environmental containment and restraint discontinued; discharged to structured residential care

*Note*: Exact calendar dates are omitted for deidentification. The table emphasizes the sequence of delusion onset, corticosteroid exposure, and olanzapine response.

Abbreviations: CBA, cell‐based assay; CSF, cerebrospinal fluid; GFAP, glial fibrillary acidic protein; IgG, immunoglobulin G; TBA, tissue‐based assay.

He was admitted under the neurology service for evaluation and treatment of suspected autoimmune encephalitis. CSF examination showed inflammatory abnormalities, including pleocytosis and oligoclonal bands. Neuroimaging was not diagnostic at the initial stage. CSF GFAP‐IgG testing performed at Gifu University supported the diagnosis of autoimmune GFAP astrocytopathy. The qualitative antibody evaluation used a cell‐based assay with HEK293 cells expressing GFAP‐alpha and a tissue‐based assay with immunohistochemistry on rat brain frozen sections; reactivity was confirmed at a 1:4 dilution by both assays.

Before corticosteroid pulse therapy or oral prednisolone, his family noticed a new persecutory delusion: he stated that someone was outside the window. This symptom was not a vague anxious impression but a concrete psychotic belief with suspiciousness. During the early neurological admission, psychiatrists assessed the emerging psychiatric symptoms through consultation. Possible hallucinatory experiences were also documented during the early psychiatric assessment, including perceiving his wife's face around the physician despite her presence beside him and statements suggesting that others were speaking ill of him. Because the sensory modality and exact interpretation of these experiences could not be definitively established, the pre‐corticosteroid persecutory delusion was considered the most robust evidence of psychosis before steroid exposure.

Intravenous corticosteroid pulse therapy was then initiated for GFAP astrocytopathy and followed by oral prednisolone; the maximum documented oral prednisolone dose was 60 mg/day. Tremor and dysarthria partially improved. However, psychotic and behavioral symptoms became clinically prominent, including persecutory ideas that money had been stolen, suspiciousness that others were watching him, grandiose ideas, marked insomnia, emotional lability, irritability, and aggression toward family members.

After repeated difficulty maintaining safety at home, he was admitted to a psychiatric ward. On admission, he showed agitation, disorganized behavior, poor impulse control, labile affect, and bilateral hand tremor. Direct mental status examination was complicated by linguistic factors because Spanish was his first language, his Japanese proficiency was limited, and his speech became almost entirely Spanish when emotionally aroused. Therefore, serial mental status examinations were supplemented by family collateral information, nursing observations, and behavioral indices such as sleep, aggression, escape behavior, need for environmental containment, and need for physical restraint.

Laboratory testing did not suggest infection, hepatic dysfunction, severe metabolic disturbance, or another acute systemic cause of psychosis. The working psychiatric diagnosis was a psychotic disorder due to another medical condition, namely, autoimmune GFAP astrocytopathy.

Prednisolone was continued because discontinuation was considered inappropriate for the underlying autoimmune neurological disease. Olanzapine was started and titrated to 15 mg/day. Despite continued prednisolone, agitation, disorganized behavior, aggression, and escape behavior improved markedly. Short‐term environmental containment and physical restraint were no longer required. Persecutory delusional preoccupation decreased, although residual grandiose delusional ideas, including claims of substantial financial resources and plans to purchase and rent apartments, persisted during family visits. He was discharged to a structured residential setting with outpatient psychiatric and neurological follow‐up.

## DISCUSSION

This case has four clinically important implications. First, it expands the psychiatric phenotype of autoimmune GFAP astrocytopathy. Previous reports have described psychiatric symptoms in GFAP astrocytopathy, but these have often been reported as broad categories such as psychiatric symptoms, delirium, cognitive impairment, apathy, anxiety, depressive symptoms, or behavioral change.^3^, ^4^, ^8^, ^9^, ^10^ Tomczak et al. reported acute delusional psychosis in a patient with GFAP autoimmunity and parkinsonism,^9^ supporting that frank psychosis can occur; however, detailed descriptions of psychotic phenomenology, relationship to corticosteroid exposure, and antipsychotic treatment response remain limited. The present patient developed a clear persecutory delusion before corticosteroid exposure, followed later by grandiosity, possible hallucinatory experiences, insomnia, irritability, and behavioral dyscontrol.

Second, the chronology strongly rules out steroid‐induced psychosis as the mechanism of onset. Corticosteroid psychiatric adverse effects are well recognized, and antipsychotics are sometimes used when corticosteroid reduction is not feasible.^13^, ^14^, ^15^, ^16^ However, a medication‐induced psychosis requires symptom onset after exposure to the medication and lack of a better medical explanation. In this case, the first concrete delusion was observed before corticosteroid pulse therapy or oral prednisolone. Moreover, the patient had subacute neurological symptoms, inflammatory CSF findings, and CSF GFAP‐IgG positivity by cell‐based and tissue‐based assays. Corticosteroids may have contributed to later insomnia or behavioral activation, but they did not cause the onset of delusional psychosis.

Third, the pathophysiology of psychotic symptoms in autoimmune GFAP astrocytopathy remains uncertain, but several mechanisms are plausible. Because GFAP is an intracellular astrocytic protein, GFAP‐IgG is generally considered a disease biomarker rather than a directly pathogenic surface antibody. Neuropathological studies suggest prominent perivascular inflammation and T‐cell‐mediated mechanisms, including CD8‐positive T‐cell involvement.^17^, ^18^ Inflammatory astrocytic dysfunction, cytokine effects, blood–brain barrier disruption, and involvement of frontolimbic or subcortical networks may therefore contribute to delusions, emotional lability, and behavioral dyscontrol. These hypotheses remain speculative in the present case because regional inflammatory activity was not directly measured.

Fourth, the therapeutic course is informative. In steroid‐induced psychosis, dose reduction or discontinuation of the offending drug is often considered, but this strategy may be unsafe when corticosteroids are required for active autoimmune neurological disease.^13^, ^14^, ^15^, ^16^ In the present case, olanzapine was markedly effective for behavioral dyscontrol even though prednisolone was continued. This response does not prove a primary psychiatric disorder; rather, it shows that symptomatic antipsychotic treatment can stabilize agitation, aggression, escape behavior, and delusional preoccupation while necessary immunotherapy is maintained. At the same time, residual grandiose delusional ideas persisted, indicating that antipsychotic response was clinically important but incomplete. This point is particularly relevant for liaison psychiatry and neurology teams.

The case has limitations. It is a single case, and formal psychosis rating scales and structured neuropsychological assessments were limited. Symptom severity and treatment response were assessed clinically using serial psychiatrist examinations, family reports, nursing observations, sleep and behavior records, and changes in the need for restraint and environmental containment. GFAP‐IgG testing was qualitative; therefore, correlations between antibody level and psychiatric severity could not be assessed. Linguistic factors may have limited direct assessment of thought content. Finally, although corticosteroids were excluded as the cause of psychosis onset, their contribution to later insomnia or behavioral activation cannot be completely ruled out.

## CONCLUSION

Frank persecutory delusions can precede corticosteroid exposure in autoimmune GFAP astrocytopathy. In such cases, steroid‐induced psychosis should not be assigned as the primary diagnosis merely because psychosis later worsens during corticosteroid therapy. The present case supports autoimmune GFAP astrocytopathy‐associated organic psychosis and demonstrates that olanzapine may markedly improve behavioral dyscontrol while prednisolone is continued for the underlying neurological disease, although residual delusional symptoms may persist.

## AUTHOR CONTRIBUTIONS

**Tomomi Sugisaki**: Conceptualization; investigation; writing—original draft. **Yoshiteru Sato**: Investigation; supervision; writing—review and editing. **Hiroaki Fujita**: Investigation; supervision; writing—review and editing. **Yasushi Kawamata**: Investigation; supervision; writing—review and editing. **Norio Sugawara**: Investigation; supervision; writing—review and editing. **Keisuke Suzuki**: Investigation; supervision; writing—review and editing. **Norio Yasui‐Furukori**: Conceptualization; investigation; writing—original draft. Hiroaki Fujita and Keisuke Suzuki contributed to the neurological evaluation and management. All authors reviewed and approved the revised manuscript.

## CONFLICT OF INTEREST STATEMENT

N.Y.‐F. reports having received honoraria for lectures from Daiichi Sankyo, EA Pharma, Eisai, Eli Lilly and Company, Hisamitsu Pharmaceutical, Janssen Pharmaceuticals, Kowa, Lundbeck, Meiji Seika Pharma, Mitsubishi Tanabe Pharma, Mochida Pharmaceutical, MSD, Ono Pharmaceutical, Otsuka Pharmaceutical, Shionogi, Sumitomo Pharma, Takeda Pharmaceutical, Tsumura, Viatris, and Yoshitomi Yakuhin; has received public research funding from the Japan Agency for Medical Research and Development (AMED), Grants‐in‐Aid for Scientific Research (KAKENHI), and Health and Labour Sciences Research Grants from the Ministry of Health, Labour and Welfare of Japan; has served in advisory or consultancy roles for EA Pharma, Bristol Myers Squibb, Boehringer Ingelheim, Janssen Pharmaceuticals, Mitsubishi Tanabe Pharma, Mochida Pharmaceutical, Sumitomo Pharma, and Otsuka Pharmaceutical; and has participated as a sub‐investigator in industry‐sponsored clinical trials conducted by EA Pharma and Bristol Myers Squibb. All other authors declare no conflicts of interest.

## ETHICS APPROVAL STATEMENT

This is a single deidentified case report. Formal ethics approval was not required according to the ethics committee of Dokkyo Medical University Hospital.

## PATIENT CONSENT STATEMENT

Written informed consent for publication was obtained from the patient or the appropriate representative.

## CLINICAL TRIAL REGISTRATION

Not applicable.

## Data Availability

Data sharing is not applicable to this article because no datasets were generated or analyzed for this case report.
